# Inhibition of CRM1 activity sensitizes endometrial and ovarian cell lines to TRAIL-induced cell death

**DOI:** 10.1186/s12964-018-0252-z

**Published:** 2018-07-04

**Authors:** François Fabi, Pascal Adam, Keven Vincent, Françis Demontigny, Sophie Parent, France-Hélène Joncas, Eric Asselin

**Affiliations:** Department of Medical Biology, Université du Québec à Trois-Rivières, 3351 boul. Des Forges, Trois-Rivières, Québec, G8Z 4M3 Canada

**Keywords:** Apoptosis, CRM1, TRAIL, p53

## Abstract

**Background:**

CRM1 enrichment has been shown to be indicative of invasive as well as chemoresistant tumors. On the other hand, TRAIL, a powerful and specific anti-tumoral agent, has yet to be used effectively to treat gynecological tumors in patients. In the present study, we examined if CRM1, a nuclear exporter capable of mediating protein transport, could be a relevant target to restore chemosensitivity in chemoresistant cells. We thus explored the hypothesis that CRM1-driven nuclear exclusion of tumor suppressors could lead to chemoresistance and that CRM1 inhibitors could present a novel therapeutic approach, allowing sensitization to chemotherapeutic agents.

**Methods:**

Ovarian cancer cell lines, as well as endometrial cancer cell lines, were treated with leptomycin B (LMB), cisplatin and TRAIL, either singly or in combination, in order to induce apoptosis. Western blot and flow cytometry analysis were used to quantify caspases activation and apoptosis induction. Immunofluorescence was used to determine nuclear localization of p53. Colony formation assays were performed to determine therapeutic effectiveness; p53 siRNA were used to establish p53 role in sensitization. Additional information from GEO database and Prognoscan allowed us to contextualise the obtained results. Finally, qRT-PCR was performed to measure apoptotic regulators expression.

**Results:**

TRAIL and LMB combination therapy lead to cleavage of caspase-3 as well as the appearance of cleaved-PARP, and thus, apoptosis. Further experiments suggested that sensitization was achieved through the synergistic downregulation of multiple inhibitor of apoptosis, as well as the activation of apoptotic pathways. p53 was enriched in the nucleus following LMB treatments, but did not seem to be required for sensitization; additional experiments suggested that p53 opposed the apoptotic effects of LMB and TRAIL. Results obtained from public data repositories suggested that CRM1 was a driver of chemoresistance and poor prognostic; DR5, on the other hand, acted as as a marker of positive prognostic.

**Conclusions:**

Taken together, our results suggest that the use of CRM1 inhibitors, in combination to chemotherapeutic compounds, could be highly effective in the treatment of gynecological malignancies.

**Electronic supplementary material:**

The online version of this article (10.1186/s12964-018-0252-z) contains supplementary material, which is available to authorized users.

## Background

The vast majority of tumors found in human cancer can be fought through the use of chemotherapeutic drugs. However, chemoresistance is a major hurdle in the treatment of cancer, especially in the case of ovarian and endometrial cancers [1, 2]. The strikingly high mortality rate observed in ovarian cancer, as well as relapsing endometrial cancer, can be explained by the onset of this type of resistance, which renders our usual drugs regimens ineffective. It is therefore crucial that we develop novel strategies to overcome chemoresistance and reinstate the effectiveness of various chemotherapeutic agents. The ability, or lack thereof, of cells to respond to chemotherapeutic agents is widely thought to be correlated to their readiness to enter the apoptotic program; this program, governed by a wide variety of both pro-survival and pro-apoptotic proteins, is largely dysregulated in tumor cells, through either mutations or functional silencing. Oncogenic mutations, either providing gain-of-function to proto-oncogenes or loss of function to tumor suppressors, are difficult to drug efficiently; the nature of the mutations often confer independence from upstream signaling or become incapable of downstream signaling. Alternatively, mislocalization of key proteins can alter their specific activity, either through substrate availability, or lack thereof, as well as turnover modulation. In this paper, we will clarify the relationship between CRM1, an ubiquitous and fundamental actor of the nuclear export machinery, and chemoresistance of gynecological malignancies. We will present results that support for the first time the effectiveness of nuclear export inhibitors in the treatment of gynecological cancers through the reinstatement of TRAIL-induced apoptosis sensitivity. Finally, we will underline the paradoxical effect of p53 localization and expression on these sensitization mechanisms.

Chromosomal maintenance 1 (CRM1) is part of a conserved superfamily of RanGTP-binding transporters that regulates and facilitates nuclear pore passage of RNAs, proteins and RNPs from the nucleus to the cytoplasm compartment [3]. CRM1 binds to leucine-rich motifs known as nuclear export signals (NES) that are present on its cargoes; CRM1 interacts with its target and then readily transports it out of the nuclear compartment. The presence of a NES on the target protein is critical for its binding and interaction with CRM1 [4]. CRM1 interaction with NES-bearing proteins is inhibited by the action of leptomycin B (LMB), a potent antifungal antibiotic produced by *Streptomyces* that displays powerful antitumor abilities, especially in the context of drug resistant cancers. This drug induces apoptosis through the inhibition of several tumor suppressors’ export-driven nuclear exclusion, thereby potentiating their action in the nucleus [1, 5–7]. p53 is a well-known tumor suppressor, considered as one of the most pivotal regulator of cell fate; interestingly, p53 localization is highly dependent on CRM1 driven export [8]. The p53 tumor suppressor is one of the most widely mutated protein in ovarian cancer, with more than 94% of high grade serous ovarian carcinomas presenting a mutated p53, 62% of which are missense mutations [9]. Serous endometrial carcinomas, part of the type 2 endometrial tumor type family, also presents a p53 mutation rate as high as 90% [10]. Multiple studies have demonstrated the potent ability of LMB to induce apoptosis in otherwise resistant cancer cells, either alone or in combination with chemotherapy, mainly through p53 stabilisation and subsequent activation [6, 11–13]. While p53 mutations generally bestows resistance to multiple type of chemotherapeutic approaches, LMB effect on apoptosis induction remains poorly understood in gynecological tumors, especially in the ovarian tumorological context presenting almost universal p53 mutations. In all cases, apoptosis can be triggered through the intrinsic or extrinsinc pathway. While the former is dependant upon DNA damage, the latter involves membrane-bound receptors activated by various ligands. Many receptors and ligands have been characterized to date, namely Fas-ligand, which uses the Fas receptor (FasR), TNFα, which uses TNF-receptor 1 (TNFR1) and TRAIL, which uses Death receptor-4 and 5 (DR4–5); all of these receptors are members of the tumor necrosis factor receptors family. They all possess an intracytoplasmic domain called the “death domain” which can, upon ligand binding, recruit intracellular adapter proteins such as FADD, which will in turn recruit procaspase-8. This adapter complex, aptly named death-inducing signaling complex (DISC), will then activate downstream caspases and initiate the execution phase of apoptosis. [14, 15]. This convergent finality of both the intrinsic and extrinsic death pathways is characterized by the cleavage and activation of caspase-3, − 6 and − 7; however, caspase-3 is widely considered as the penultimate executioner of the apoptotic program. While gynecological malignancies will often develop cisplatin resistance at later stages [16], most of them are almost completely resistant to TRAIL-induced apoptosis, partly owing to abnormal FLIP expression [17–20]. Many proteins also oppose the TRAIL-induced apoptotic process, such as XIAP, which inhibits signal transduction as well as caspases activation and MCL-1, which counteracts the ability of Bcl-2 family proteins to induce cytochrome C release [14, 15]. While early clinical trials hinted at TRAIL potential as a novel, tumor-specific therapy, this enthusiasm was impeded by the increasingly clear inability of TRAIL single therapy to reliably induce therapeutic response [17]. Par-4, a tumor suppressor first discovered in apoptotic prostatic cancer cells [21] and ubiquitously expressed throughout the body, is responsible for apoptosis induction in multiple cell types [22–27]. Undoubtedly, Par-4 most interesting ability resides in its capacity to induce death selectively in tumor cells, sparing normal cells from cellular suicide, in a manner reminiscent of TRAIL specificity [10, 11]. We have also recently reported that Par-4 is cleaved by caspase-3 at EEPD(131)↓G, generating a 25 kDa fragment (cleaved-Par-4) that is capable of inducing apoptosis and that this cleavage was inhibited by XIAP activity [28]. In this research we have studied the effect of LMB on chemosensitization of gynecological cancers as well as the role of CRM1 in this process. We have also assessed the effectiveness of combination therapy of LMB and chemotherapeutic drugs that induce enhanced cell death in chemoresistant cancer cell lines as well as the role of p53 localization in this mechanism. Finally, we demonstrated the ability of LMB to reliably and powerfully sensitize multiple cell types, presenting both mutated and wild-type p53, to TRAIL-induced apoptosis in a p53-independent manner.

## Methods

### Cell lines and reagents

KLE, OVCAR-3 and SKOV-3 cell lines were purchased from ATCC (Manassas, VA, USA). HIESC cells were graciously offered by Michel A. Fortier (Université Laval, Québec, Canada). A2780 and A2780CP were kindly provided by Dr. G. Peter Raaphorst (Ottawa regional cancer center, Ottawa, Canada). Ishikawa cells were kindly provided by Dr. Sylvie Mader (Université de Montréal, Montréal, Canada). ECC-1 cells were kindly provided by Nicolas Gévry (Université de Sherbrooke, Sherbrooke, Canada). The chosen cell lines allow us to mimic multiple characteristics of gynegological cancers by recapitulating main mutations and molecular hallmarks found in patients. Ishikawa are a well differentiated, ERα-positive cell line derived from a low-grade adenocarcinoma; Ishikawa are PTEN-null and express mutated p53 [29–31]. ECC-1 are a well differentiated, ERα-positive cell line derived from a low-grade adenocarcinoma; ECC-1 are PTEN-null and presents no p53 mutations [32–35]. KLE are a poorly differentiated, ERα-negative cell line derived from high-grade adenocarcinoma; KLE express wild-type PTEN and mutated p53 [36–38]. A2780 are a poorly differentiated, ERα-negative cell line derived from high-grade ovarian adenocarcinoma; A2780 express mutated PTEN and wild-type p53 [39–41]; A2780CP are very similar, having been generated from the former cell line, but express mutated p53 [39, 42]. OVCAR-3 are a poorly differentiated, ERα-positive cell line derived from a high-grade ovarian adenocarcinoma; OVCAR-3 express wild-type PTEN and mutated p53 [39, 41, 43]. Finally, SKOV-3 are a poorly differentiated, ERα-positive cell line derived from high-grade ovarian adenocarcinoma; SKOV-3 express wild-type PTEN and are p53-null [41, 44]. All the antibodies, as well as leptomycin B, were obtained from Cell Signaling Technology (Danvers, MA, USA) except for the anti-rabbit secondary antibody used for western blotting (Bio-Rad Laboratories, Hercules, CA, USA) and for the Alexa Fluor 488 tagged anti-rabbit secondary antibody, which was obtained from Thermo Fisher Scientific Inc. (Waltham, MA, USA). Recombinant TRAIL, Annexin V/PI used for flow cytometry experiments and siRNAs were procured from Thermo Fisher Scientific Inc. (Waltham, MA, USA). X-2 transfecting agent was procured from Mirus (Madison, WI, USA). Cisplatin was purchased from Sigma-Aldrich (St. Louis, MO, USA).

### Flow cytometry

FITC annexin V/dead cell apoptosis kit was used according to the manufacturer’s instructions. Briefly, the treated cells were collected, washed with PBS, and then diluted in 1× annexin binding buffer (100 μL). For each sample, 5 μL of annexin V and 1 μL of propidium iodide were added to the cell suspension and then incubated 15 min at room temperature. After incubation time, an additional 100 μL of the annexin binding buffer was added to each sample for a total of 200 μL. Samples were analyzed (6000–10,000 events) using a Beckman Coulter flow cytometer Cytomics FC500 (Beckman Coulter, Mississauga, Ontario, Canada).

### MTT assays

Briefly, plates were seeded with 180 μL of normal and cancer cells in suspension (for HIESC, 14000; Ishikawa, 16,000; ECC-1, 14,000; A2780/CP, 16000; OVCAR-3, 16,000) in medium using 96-wells plates. Plates were incubated at 37 °C, 5% CO_2_ for 24 h. TRAIL, cisplatin and leptomycin B were diluted in fresh medium, serially diluted and added to the plates to obtain the final indicated concentration. Cell were then incubated for another 24 h after which 10 μL of 3-(4,5-dimethylthiazol-2-yl)-2,5-diphenyltetrazolium bromide (MTT) (5 mg/mL in PBS) were added to the wells. Four hours later, 100 μL of the solubilization solution (10% sodium dodecyl sulfate (SDS) in 0,01 M HCl) were added and the plates incubated overnight (37 °C, 5% CO_2_). The optical density was read using a FluoStar Optima BMG (BMG Labtech Inc., Durham, NC, USA) at 565 nm. Each experiments were performed in duplicate on the same plate.

### Western blot analysis

After the end of the treatment period or transfection time, both floating and attached cells were collected and cell lysate was done using cold radioimmunoprecipitation assay lysis buffer containing protease inhibitors (Complete; Roche Applied Science, Indianapolis, IN, USA), followed by three freeze–thaw cycles. Proteins were measured using the Bio-Rad DC protein assay. Western blotting was performed following a classical protocol. Appropriate peroxidase-conjugated secondary antibodies were used, and the blot was developed using SuperSignal West Femto substrate (Thermo Scientific, Rockford, IL, USA), as described by the manufacturer, using a cooled CCD camera (UVP System). The shown results are representative of at least three independent experiments.

### Colony formation assays

Cells were plated at a confluence of 2000 cells per well in a 6 wells plate and grown for 24 h. Cells were then treated for 24 h after which the media was replaced. Cells were allowed to grow for ten days and media was replaced every 5 days. After 10 days, cells were washed with PBS and fixed in ice-cold formalin for 10 min. After fixation, colonies were colored with Giemsa Stain 0.4% for 5 min. Plates were then washed with running water, allowed to dry and colonies were photographed using a cooled CCD camera. Images were quantified using the ColonyArea software [45].

### RT-qPCR

To measure the transcripts levels, total RNA was isolated from cells using RNeasy Mini Kit from QIAGEN (Mississauga, ON, Canada). Total RNA (1 μg) was subjected to reverse transcription using qScript cDNA Supermix (Quanta Biosciences, Gaithersburg, MD) as described by the manufacturer’s instructions. The reverse-transcribed RNA was then amplified by PCR using specific primers. The expression of DR4, DR5, DcR1, DcR2, PUMA, p21 and p27 were measured through the use of specific primers detailed in Table 1. Each reaction mixture (final volume, 25 μL) were performed using Perfecta SYBR Green Supermix Low Rox (Quanta Biosciences, Beverly, MA, USA) according to manufacturer protocol and quantified using a Mx3000P system (Agilent Technologies, Mississauga, Ontario, Canada). For each gene target, a standard curve was generated to determine the efficiency of the reaction, and the Pfaffl analysis method was used to measure the relative quantity of gene expression. Each real time PCR was performed in duplicates and results were drawn from at least three independent experiments. 18S was used as a reference gene based on its stable expression in all cells and between all treatments. The Pfaffl method of quantification was used to measure relative expression.

**Table 1:** Primers sequence

Targeted gene	5’- Forward primer − 3’	5’- Reverse primer − 3’
DR4	cagagggatggtcaaggtcaagg	ccacaacctcagccgatgc
DR5	cgctgcaccaggtgtgatt	gtgccttcttcgcactgaca
DcR1	accaacgcttccaacaatgaa	ctagggcacctgctacacttc
DcR2	gttggcttttcatgtcggaaga	cccaggaactcgtgaaggac
PUMA	acctcaacgcacagtacgag	cccatgatgagattgtacagga
p21	ctggagactctcagggtcgaaa	gattagggcttcctcttggagaa
p27	ggcctcagaagacgtcaaac	acaggatgtccattccatga
18 s	tggtcgctcgctcctctccc	cagcgcccgtcggcatgtat

### siRNA and transfections

For silencing of p53 expression, cells were seeded in 6-well plates (∼6 × 10^5^ cells per well) and reversed transfected with 50 nM of p53 siRNA (5’- GGAUUUCAUCUCUUGUAUAtt − 3) or control scrambled siRNA. In order to perform the reverse transfection, we used the Mirus X-2 transfection reagent in accordance with the manufacturer’s instructions. Following reverse transfection, cells were grown for 24 h and the media was then replaced; treatments and subsequent analyses were then performed as described before.

### Immunofluorescence

Cells were treated as described above and were grown in 6-well plates containing sterile coverslips. On the day of analysis, cells were fixed with 4% paraformaldehyde for 10 min, and permeabilized for 10 min using 0.1% Triton X-100 in 0.1% sodium citrate at room temperature. After blocking with 4% normal goat serum blocking for 1 h, cells were incubated with primary antibody at a concentration of 1μg/mL or isotypic control antibody for 1 h. After incubation with primary antibody, cells on the coverslips were washed three times with PBS and then incubated with Alexa Fluor 488 secondary antibodies (1:800 dilution) for 30 min at room temperature in dark conditions. Cells were counterstained with Hoechst 33,248 (0.25 μg/ml) for 5 min, and slides were mounted using Slowfade gold antifading reagent (Invitrogen) and viewed under a Leica TCS SP8 confocal microscope, using a 63× immersion lens (Leica Microsystems, Concord, Ontario, Canada).

### Statistical analyses

Statistical analysis was done by one-way analysis of variance with Tukey’s post hoc test or Student’s *t*-test where appropriate. Combination therapy data were subjected to 2-way ANOVA. Interaction rating emanating from the 2-way ANOVA was used to determine synergism between studied drugs [46, 47]. Generally, the interaction quantified in a 2-way ANOVA can be compared to a null hypothesis test (no direct interaction, which is a fundamentally similar effect to additivity, and thus, absence of synergism). From this premise, highly significant interaction between drugs effect on cell death suggest form of synergism. When 2-way ANOVA failed to show synergistic effect, differences between experimental groups were determined by t-test. Statistical significance was accepted when *P* < 0.05. *P < 0.05; **P < 0.01; ***P < 0.001. All analysis was performed using GraphPad PRISM software, version 3.03 (GraphPad Software, Inc., La Jolla, CA, USA).

## Results

### Leptomycin B combination therapy significantly reduces cell viability in a tumor specific manner (Fig. 1)

**Fig. 1:**
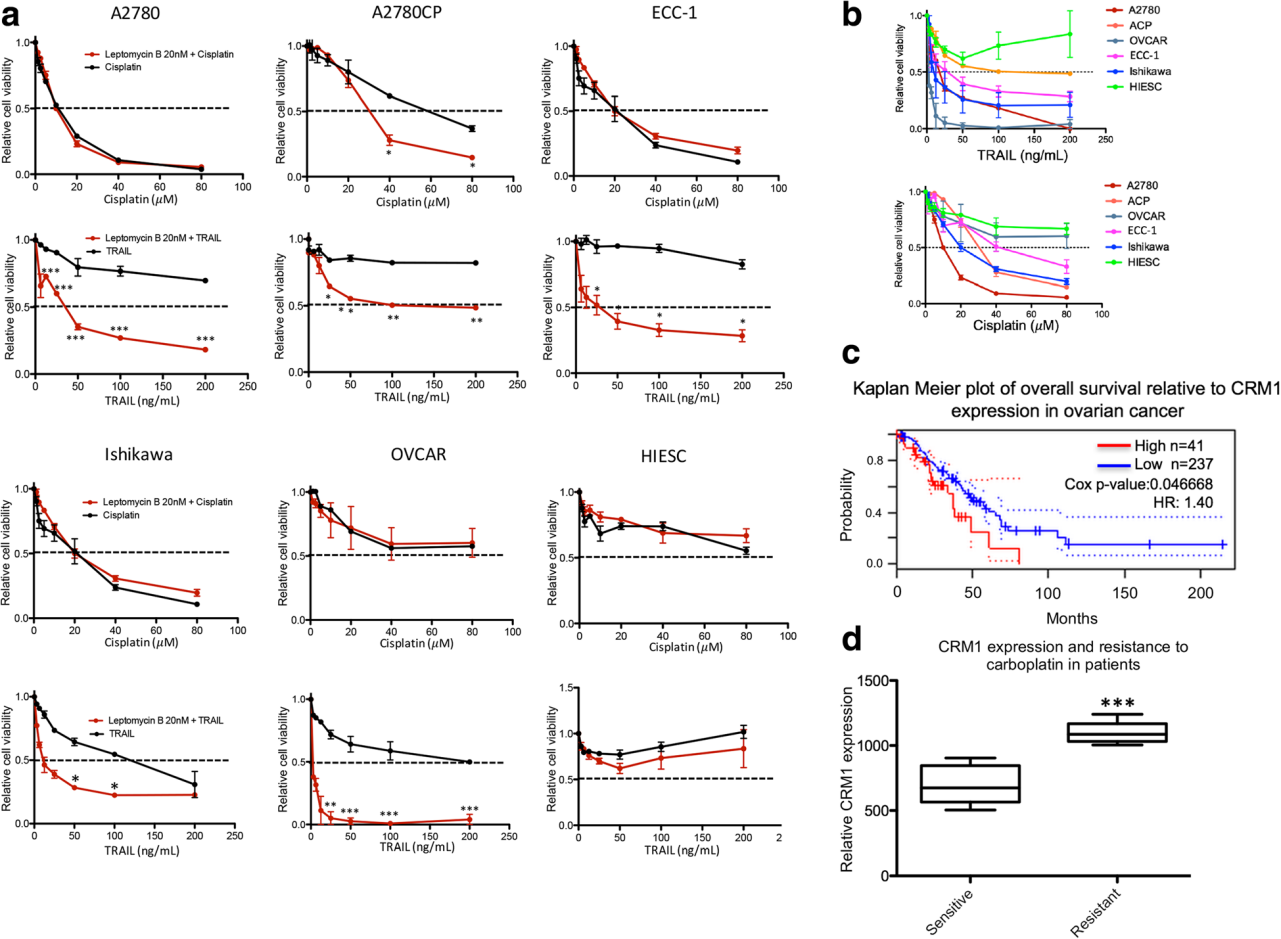
Leptomycin B combination therapy significantly reduces cell viability in a tumor specific manner. **a** Studied cell lines were treated with increasing concentration of cisplatin (0-80 μM) and TRAIL (0-200 ng/mL) in presence or absence of leptomycin B (20 nM) for 24 h. The MTT was then used to determine the resultant changes in cell viability. Results shown are representative of three independent experiments. **b** Comparison between the combined therapy results of every cell line for both chemotherapeutic agents. **c** Kaplan Meier plot showing the significantly increased survival rate found in ovarian cancer patients presenting low level of CRM1 expression; obtained from dataset GSE9891/235927_at **d** Box plot illustrating the significantly increased CRM1 expression in the context of carboplatin-resistant patient ovarian tumor samples; obtained from dataset GDS1381/37729_at. Except for c, in which n number is indicated specifically, all data are means ± SEM of three independent experiments. *, *p* < 0.05; **, *p* < 0.01; ***, *p* < 0.001

In order to determine the effect of LMB combination therapy with either cisplatin or TRAIL, we conducted cell viability experiments using the MTT assay. Cells were treated either with a single agent, cisplatin or TRAIL, or with a combination of either agent with LMB (Fig. 1a). Our results confirmed the previously obtained data, demonstrating that LMB significantly sensitized A2780CP cells to the cytotoxic effects of cisplatin; however, high concentrations of cisplatin alone showed the ability to reduce cell viability in most cell lines. Strikingly, TRAIL, even at high concentration, proved ineffective in reducing cell viability in almost all cell lines. However, again in agreement with previously obtained results, a significant sensitization effect was observed in the case of LMB concomitant treatment with TRAIL, confirming LMB ability to enhance TRAIL inhibitory effect on cell viability. When compared together, we also observed that the combined treatment, both in the case of LMB and cisplatin as well as LMB and TRAIL, seemed to have an almost imperceptible effect on human immortalized endometrial stromal cells (HIESC); considering that HIESC cells are transformed, non-malignant cells, this result suggest that the combination of LMB with chemotherapeutic agents could potentially exert a selective cytotoxicity, further increasing its potential therapeutic value (Fig. 1b). In order to explore the clinical implications of CRM1 expression in ovarian cancer progression and contextualise our results, we used PrognoScan [48], an online tool capable of correlating patients prognosis with gene expression by systematically mining public databases. Using this tool, we determined the role of CRM1 expression on overall survival of ovarian cancer patients. The results obtained from the dataset [49] showed that patient with high expression of CRM1 had a worst overall survival time when compared to low expressing ones (*n* = 278, HR: 1.40, Cox *p*-value:0.046668) (Fig. 1c). A second data set, obtained from GEO database, compared three ovarian cancer patients presenting carboplatin sensitivity with three resistant patients. The results found in this dataset show a clear and significant correlation (*p* < 0.001) between relative CRM1 mRNA expression and carboplatin resistance, strongly supporting the idea that CRM1 could act as a driver of chemoresistance (Fig. 1d). Taken together, the obtained results suggest that CRM1 could be a potential driver of chemoresistance and that drugs inhibiting its action, such as LMB, could act as potential therapeutic target for ovarian cancer combination therapy.

### Combination of cisplatin or TRAIL with leptomycin B synergistically induces apoptosis induction in endometrial cancer cell lines (Fig. 2)

**Fig. 2:**
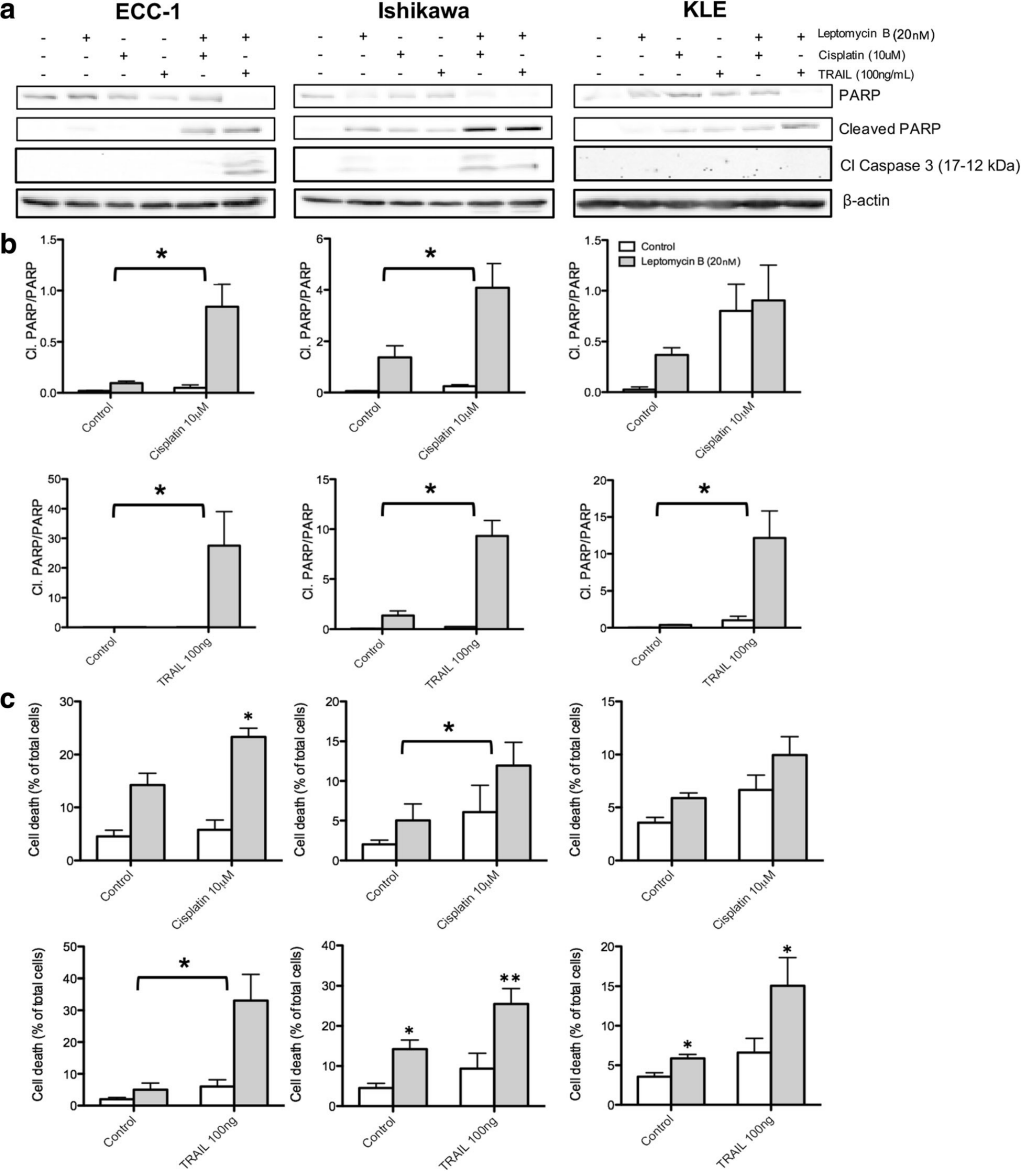
Combination of cisplatin or TRAIL with leptomycin B synergistically induces apoptosis induction in endometrial cancer cell lines. **a** Endometrial cancer cell lines were treated with leptomycin B (20 nM), cisplatin (10 μM), TRAIL (100 ng/mL) or a combination of leptomycin B with cisplatin or TRAIL for 24 h. Western blot was performed using relevant antibodies and β-Actin was used as a loading control. Results shown are representative of three independent experiments. **b** Densitometric analysis of PARP cleavage followed by 2-way ANOVA analysis; bracket indicate when interaction was statistically significant. **c** Flow cytometry analysis was performed on the cells by staining with annexin V/PI and the levels of cell death was measured; cells stained with annexin V and/or PI were used to determine the relative quantification of cell death. 2-way ANOVA was performed on the data; brackets indicate when interaction was statistically significant. All data are means ± SEM of three independent experiments. *, *p* < 0.05; **, *p* < 0.01

The previously obtained data suggested a key role for CRM1 in the chemoresistance gynecological tumors; we thus decided to screen endometrial cancer cell lines in an effort to better our understanding of these intrinsically resistant tumors [2]. We used either LMB (20 nM), cisplatin (10 μM), TRAIL (100 ng/mL) or a combination of LMB with cisplatin or TRAIL. The dosage used was determined according to concentrations used in our previous publications [50–52] as well as recent literature [6, 53, 54]. Results show that the use of any single agent failed to induce caspase-3 cleavage, with the exception of LMB in Ishikawa cells. However, combination of cisplatin and LMB treatment was successful in inducing caspase-3 cleavage in the observed cell lines, especially in the case of Ishikawa cell line where the 21 kDa as well as 17-12 kDa cleavage products can be observed in the LMB and cisplatin combined treatment. Similarly, combination of TRAIL with LMB induces the emergence of a 21 kDa caspase-3 precursor fragment as well as fully activated 12-17 kDa cleavage products in endometrial cell lines ECC-1 and Ishikawa; KLE cells did not display cleaved caspase-3 (Fig. 2a). These results indicate that combination therapy allows enhanced caspase-3 activation and suggest the subsequent induction of apoptosis. We then quantified the cleavage of PARP, a protein targeted by caspase-3 during apoptosis induction. Every examined cell line showed minute amount of PARP cleavage in response to cisplatin-only treatment. The use of LMB, however, increased PARP cleavage in both ECC-1 and Ishikawa cell lines in response to cisplatin. Similarly, TRAIL-only treatments failed to induce PARP cleavage in all the tested cell lines. The use of LMB, however, sensitized all three cell lines to TRAIL. Densitometric quantification coupled with two-way ANOVA statistical analysis revealed that the increase of PARP cleavage resulting from the combination of either LMB and cisplatin (ECC-1 and Ishikawa) or LMB and TRAIL (ECC-1, Ishikawa and KLE) was synergistic (Fig. 2b). Further analysis using annexin V/PI flow cytometry assays show similar results (Fig. 2c); however, only the combined use of LMB and cisplatin (Ishikawa) as well as LMB and TRAIL (ECC-1) displayed synergistic effects. These results suggest that the combined use of LMB sensitizes, in a significantly synergistic fashion, endometrial cell lines to TRAIL induced cleavage of PARP and subsequent induction of apoptosis.

### Combination of cisplatin or TRAIL with leptomycin B synergistically induces apoptosis induction in ovarian cell lines (Fig. 3)

We repeated the previous experiments in order establish whether the effects observed in endometrial cell lines could be duplicated in ovarian cell lines. It is well known that inherent resistance to TRAIL-induced apoptosis arises in multiple ovarian carcinoma cell lines, through still poorly described mechanisms [17, 18]; we thus hypothesized that LMB combination therapy could alleviate this therapeutic hurdle, as we previously demonstrated in endometrial cell lines. In order to study the effect of LMB combination treatments on the induction of apoptosis in ovarian cancer, we used cell lines SKOV-3 and OVCAR-3, both models being extensively used in the litterature. We also included A2780 and A2780CP cell lines in the study considering their fundamental homology; A2780CP was produced from A2780 through successive passages in presence of constant sub-lethal concentration of cisplatin, which mimics the stochastic model of clonal selection and tumor evolution observed in ovarian cancer chemotherapeutic resistance acquisition.

**Fig. 3:**
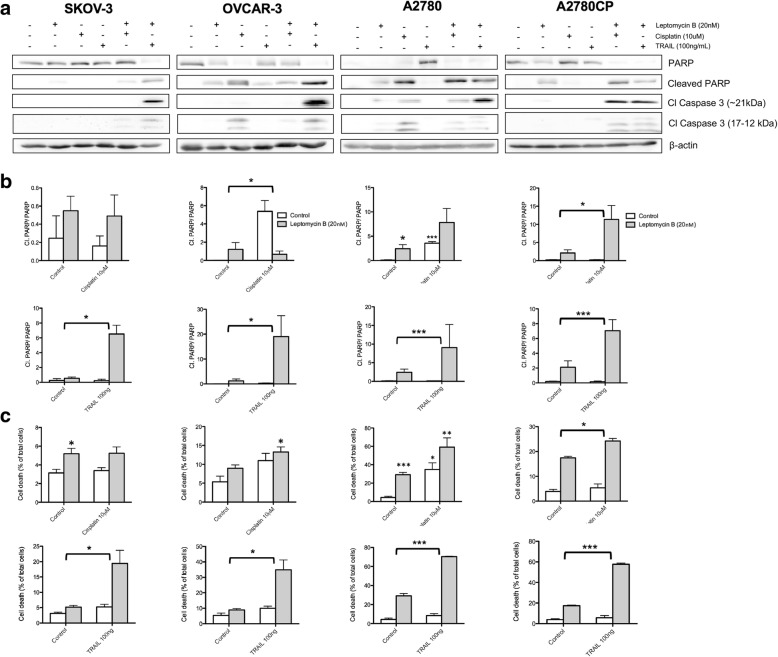
Combination of cisplatin or TRAIL with leptomycin B synergistically induces apoptosis induction in ovarian cell lines. **a** Ovarian cancer cell lines were treated with leptomycin B (20 nM), cisplatin (10 μM), TRAIL (100 ng/mL) or a combination of leptomycin B with cisplatin or TRAIL for 24 h. Western blot was performed using relevant antibodies and β-Actin was used as a loading control. Results shown are representative of three independent experiments. **b** Densitometric analysis of PARP cleavage followed by 2-way ANOVA analysis; bracket indicate when interaction was statistically significant. **c** Flow cytometry analysis was performed on the cells by staining with annexin V/PI and the levels of cell death was measured; cells stained with annexin V and/or PI were used to determine the relative quantification of cell death. 2-way ANOVA was performed on the data; brackets indicate when interaction was statistically significant. All data are means ± SEM of three independent experiments. *, *p* < 0.05; **, *p* < 0.01; ***, *p* < 0.001

We treated all cell lines with aforementioned drug regimens and then quantified caspase-3 and PARP cleavage (Fig. 3a). OVCAR-3 cells showed some measure of sensitivity to every single agent treatments as suggested by the cleavage of PARP; however, neither OVCAR-3 nor SKOV-3 displayed increased sensitivity to combined LMB and cisplatin treatment. The combination of cisplatin or TRAIL with LMB induced caspase-3 cleavage products observable at 21 kDa as well as 17-12 kDa in SKOV-3 cells; OVCAR-3 cells, on the other hand, showed such cleavage in the case of cisplatin single therapy. In accordance to their cisplatin-sensitive phenotype, cisplatin single agent therapy generated caspase-3 cleavage products observable at 21 kDa as well as 17-12 kDa in A2780 cells; however this effect was not observable in the case of TRAIL single-agent treatment. A2780CP cells, being robustly cisplatin resistant, showed no such caspase cleavage in the case of cisplatin single therapy; the use of LMB restored the ability of both chemotherapeutic agents to induce caspase-3 cleavage. In all cases, TRAIL single agent treatment failed to induce caspase-3 cleavage, an effect that was ubiquitously reversed upon combination with LMB. Densitometric quantification coupled with two-way ANOVA statistical analysis revealed that the increase of PARP cleavage resulting from the combination of LMB and TRAIL was synergistic in all cell lines (Fig. 3b); the combination of cisplatin and LMB showed no such synergistic effect, except in the case of A2780CP. Flow cytometry assays using AnnexinV/PI confirmed these results and as well as the synergistic nature of LMB and TRAIL combination treatment efficiency in inducing apoptosis (Fig. 3c). Taken together, these results strongly indicate the capacity of LMB to synergistically act with TRAIL to induce cell death through apoptosis in ovarian cell lines, as well as reversing the acquired resistance to cisplatin exhibited by A2780CP cells.

### Combination of TRAIL and leptomycin B synergistically induces extrinsic and intrinsic apoptotic programs in a p53-independent manner (Fig. 4)

**Fig. 4:**
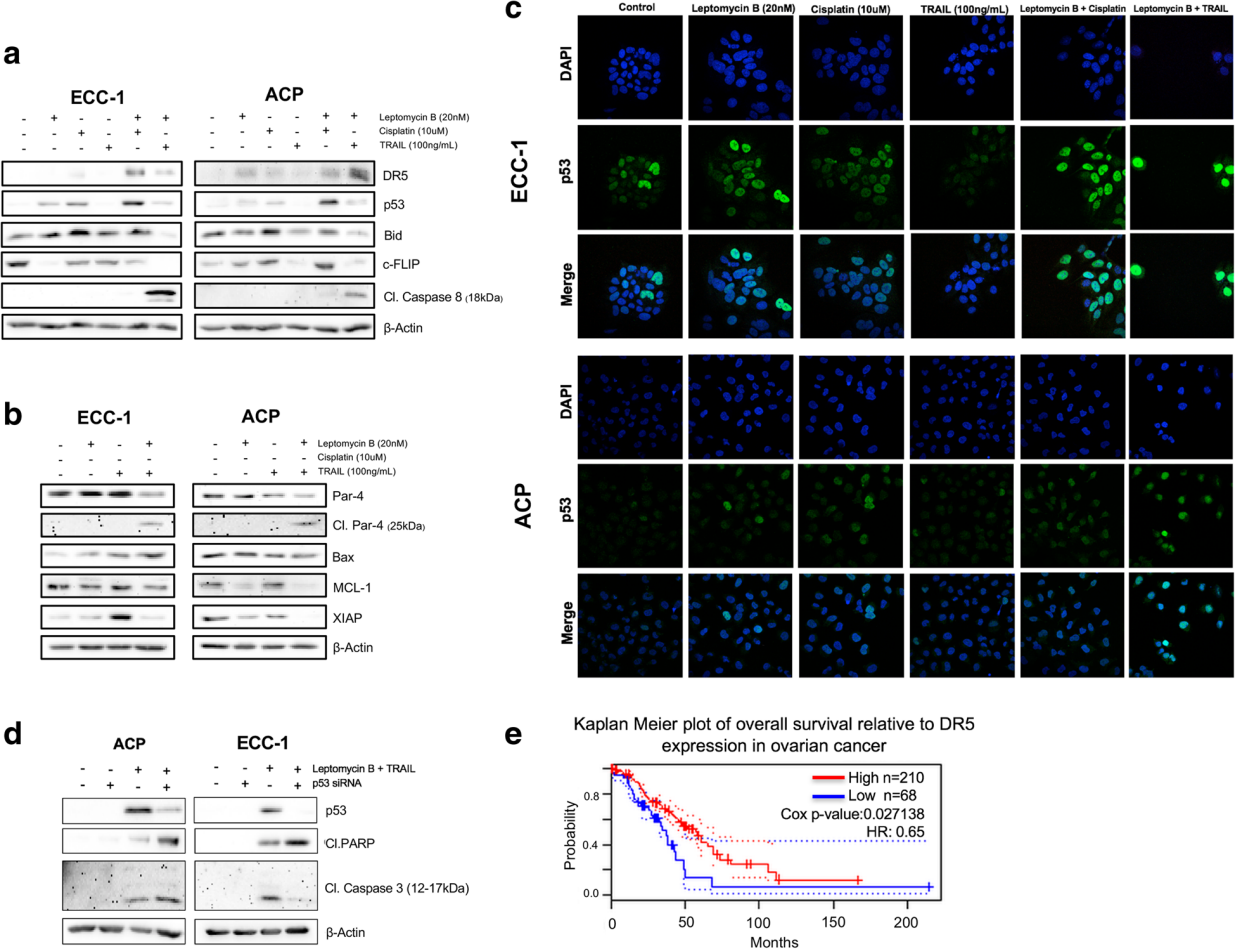
Combination of TRAIL and leptomycin B synergistically induces extrinsic and intrinsic apoptotic programs in a p53-independent manner. **a** ECC-1 and A2780CP cell lines were treated with leptomycin B (20 nM), cisplatin (10 μM), TRAIL (100 ng/mL) or a combination of leptomycin B with cisplatin or TRAIL for 24 h. Western blot was performed using relevant antibodies and β-Actin was used as a loading control. Results shown are representative of three independent experiments. **b** ECC-1 and A2780CP cell lines were treated with leptomycin B (20 nM), TRAIL (100 ng/mL) or a combination of leptomycin B with TRAIL for 24 h. Western blot was performed using relevant antibodies and β-Actin was used as a loading control. Results shown are representative of three independent experiments. **c** Immunofluorescence experiments were conducted in order to determine the effect of the previous treatments on p53 subcellular localization **d.** ECC-1 and A2780CP cell lines were reverse transfected with a p53 siRNA and then treated a combination of leptomycin B (20 nM) and TRAIL (100 ng/mL) for 24 h. Western blot was performed using relevant antibodies and β-Actin was used as a loading control. Results shown are representative of three independent experiments. **e** Kaplan Meier plot showing the significantly increased survival rate found in ovarian cancer patients presenting high level of DR5 receptors; obtained from dataset GSE9891/209294_x_at

Considering the previously obtained results, we endeavored to shed some light on the molecular mechanisms responsible for cells sensitization to TRAIL by LMB. We decided to conduct all subsequent experiments using the A2780CP ovarian cancer cell line as well as the ECC-1 endometrial cancer cell lines as they, respectively, are highly relevant models to both classical manifestation of these cancers; A2780CP is an epithelial, hormone independent, robustly cisplatin-resistant cell line presenting p53 mutations; on the other hand, ECC-1 is an epithelial, hormone responsive, mildly cisplatin-resistant cell line presenting PI3K/Akt amplifications and PTEN deletion. We first measured by Western Blot the protein level of multiple regulators of apoptosis in response to single agent treatments as well as combined treatments. We thus treated the cells with either LMB (20 nM), cisplatin (10 μM), TRAIL (100 ng/mL) or a combination of LMB with cisplatin or TRAIL. Results showed that in both studied cell lines, the combination of LMB and TRAIL allowed Bid cleavage, an upregulation in DR5 expression as well as p53, downregulation of c-FLIP and the full cleavage of caspase-8 (Fig. 4a). The densitometric analysis of these results can be found in Additional file 1: Figure S1. Further experiments involving solely LMB and TRAIL allowed us to more thoroughly characterize the modulation of key regulators of apoptotic dynamics. Firstly, in both cell lines, only the combination of LMB and TRAIL allowed the appearance of cleaved Par-4. This was accompanied by an abrogation of XIAP expression in A2780CP; alternatively, we observed a reversal of XIAP expression induced by TRAIL in ECC-1 when using a combination of LMB and TRAIL. Finally, the combination of LMB and TRAIL also increased Bax protein levels in ECC-1; this was not observable in A2780CP cells. Finally, LMB was found to downregulate MCL-1 protein levels in A2780CP when used singly as well as in combination TRAIL; this was not the case in ECC-1 cells (Fig. 4b). Considering that one of LMB most well-known mechanism of action is through the inhibition of tumor suppressors nuclear export, we also investigated the subcellular localization of p53 following the aforementioned treatments. Our results showed that the combination of LMB and TRAIL promoted robust localization of p53 to the cell nucleus (Fig. 4c). These observations suggested that p53 might be partly responsible for the sensitization effect of the combined treatments, as was hinted in multiple other publications. However, considering that TRAIL canonically induces death in a p53-independent manner and that ovarian cancer, as well as recurrent endometrial cancer, presents extensive p53 mutation profiles, we examined the effect of p53 knockdown on the induction of apoptosis in the context of LMB and TRAIL combined treatments (Fig. 4d). A2780CP cells and ECC-1 cells were reversed transfected with p53 siRNA and were subjected to a combined treatment of LMB (20 nM) and TRAIL (100 ng/mL). Indeed, the obtained results showed that p53 knockdown resulted in a significant increase in PARP cleavage in both cell lines. However, while A2780CP cells showed a slight increase in cleaved caspase-3, ECC-1 showed a drastic decrease in the processed form of the protease. Finally, we investigated the clinical relevance of elevated LMB-induced DR5 expression through Prognoscan dataming. Our results showed that high expression of DR5 (TNFRSF10B) was significantly associated with improved overall survival in patients with ovarian cancer (*n* = 278, HR: 0.65, Cox *p*-value: 0.027138) (Fig. 4e). Altogether, our results suggest that the combination of LMB and TRAIL allows for the upregulation of crucial inducer of apoptosis, Bid cleavage and downregulation of antiapoptotic proteins; interestingly, our results suggest that p53 is not required and seemingly opposes the occurrence of these events.

### Combination of TRAIL and leptomycin B significantly reduces tumor cells ability to clonally proliferate in a p53 independent manner (Fig. 5)

**Fig. 5:**
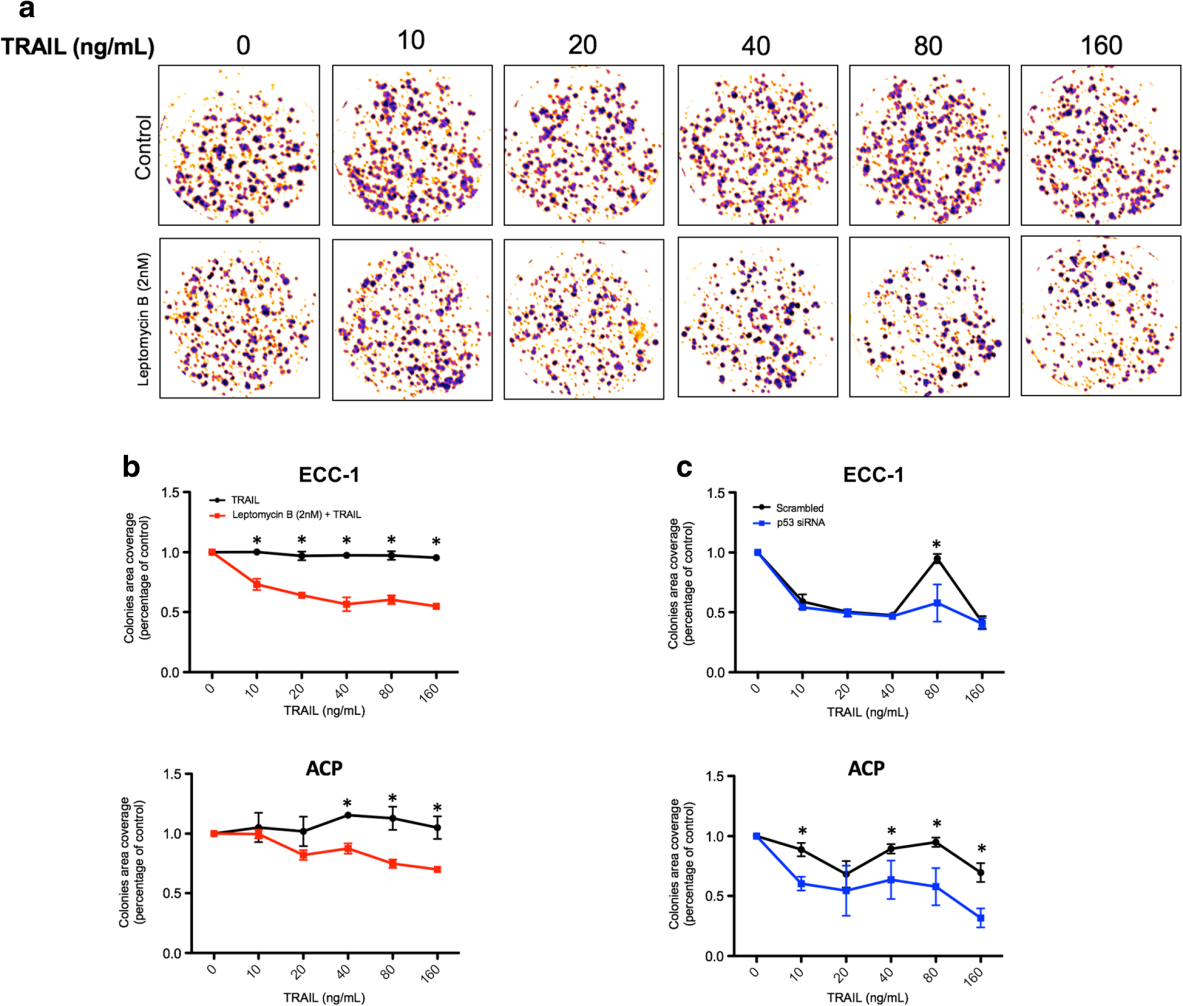
Combination of TRAIL and leptomycin B significantly reduces tumor cells ability to clonally proliferate in a p53 independent manner. **a** Studied cell lines were treated with increasing concentration of TRAIL (0-160 ng/mL) in presence or absence of leptomycin B (2 nM) for 24 h and grown for 10 days. The effect on colony formation is quantified using the densitometric map obtained following the ColonyArea software methodology. Results shown are representative of three independent experiments. **b** Comparison in colony coverage between single agent therapy and combined therapy. **c** Comparison of the effect of combined therapy in the context of p53 knockdown. All data are means ± SEM of three independent experiments. *, *p* < 0.05

In order to inquire the long-term effect of the previously demonstrated synergism between LMB and TRAIL on cell viability and induction of apoptosis, we performed clonogenic assays. This measure presents a high clinical value, considering the biological context of tumor progression. Additionally, performing a clonogenic assay allowed us to more closely mimic the longstanding effect of a single combined, lower concentration treatment on a cellular population in order to simulate more powerfully a possible future therapeutic context. Preliminary experiments suggested that LMB, used singly possessed an IC50 of ~ 4 nM in A2780CP and ECC-1 cell lines (data not shown). Considering that the cells were subjected to the treatment for 24 h and then allowed to grow for 10 days in the absence of LMB, this result suggests that LMB, even at low concentration, strongly reduce cell viability. Building on these results, we decided to use a concentration of 2 nM for the following experiments; in both cell lines, this concentration showed to have almost no effect on cell proliferation, thus enabling us to truly observe the sensitizing effect of LMB even at minimal concentrations. Cells were subjected to increasing concentrations of TRAIL, either in the presence or absence of leptomycin (2 nM). Using the ColonyArea plugin [45], we measured the pixel intensity of the obtained colonies and quantified the results (Fig. 5a). Our results showed a significant sensitization of both A2780CP and ECC-1 cells to very low concentrations of TRAIL. In the case of ECC-1, the LMB treatment allowed a significant decrease in cell proliferation potential at a concentration of as low as 10 ng/mL of TRAIL; a similar effect was observed in A2780CP cells, with a significant decrease in cell proliferation potential found at 40 ng/mL of TRAIL and higher. It is interesting to note that without LMB, TRAIL seemed to bolster proliferation in A2780CP cells, possibly through a positive feedback loop gained by the selection of resistant cells by the single agent treatment. Together, these results clearly demonstrate the ability of LMB to strongly sensitize cells to the proliferative inhibition effect of TRAIL (Fig. 5b). We also investigated the role of p53 in this process; considering that LMB effect is widely considered to be dependent upon p53 nuclear accumulation, it was, in our opinion, crucial to demonstrate the impact of p53 depletion on the observed sensitization effect. We performed experiments using identical concentrations as described above; however, upon plating, cells were reversed transfected using either p53 siRNA or scrambled siRNA as control. Cells were then treated with 2 nM of LMB as well as increasing concentrations of TRAIL. In accordance with our previously obtained data, the gathered results showed that p53 depletion significantly sensitized A2780CP cells to a concentration of 10 ng/mL of TRAIL and higher. On the other hand, p53 depletion showed almost no effect on ECC-1 cells, but showed a significant sensitization effect at 80 ng/mL of TRAIL (Fig. 5c). These results suggested that LMB was capable of sensitizing cells to the anti-proliferative effects of TRAIL. Interestingly, p53 appeared to oppose this sensitization mechanism, both in the context of a cell line presenting mutated p53 (A2780CP) as well as wild-type p53 (ECC-1).

### Leptomycin B, both singly and in combination with chemotherapeutic agents, modulates the expression of crucial apoptotic pathway genes in a cell-type specific manner (Fig. 6)

**Fig. 6:**
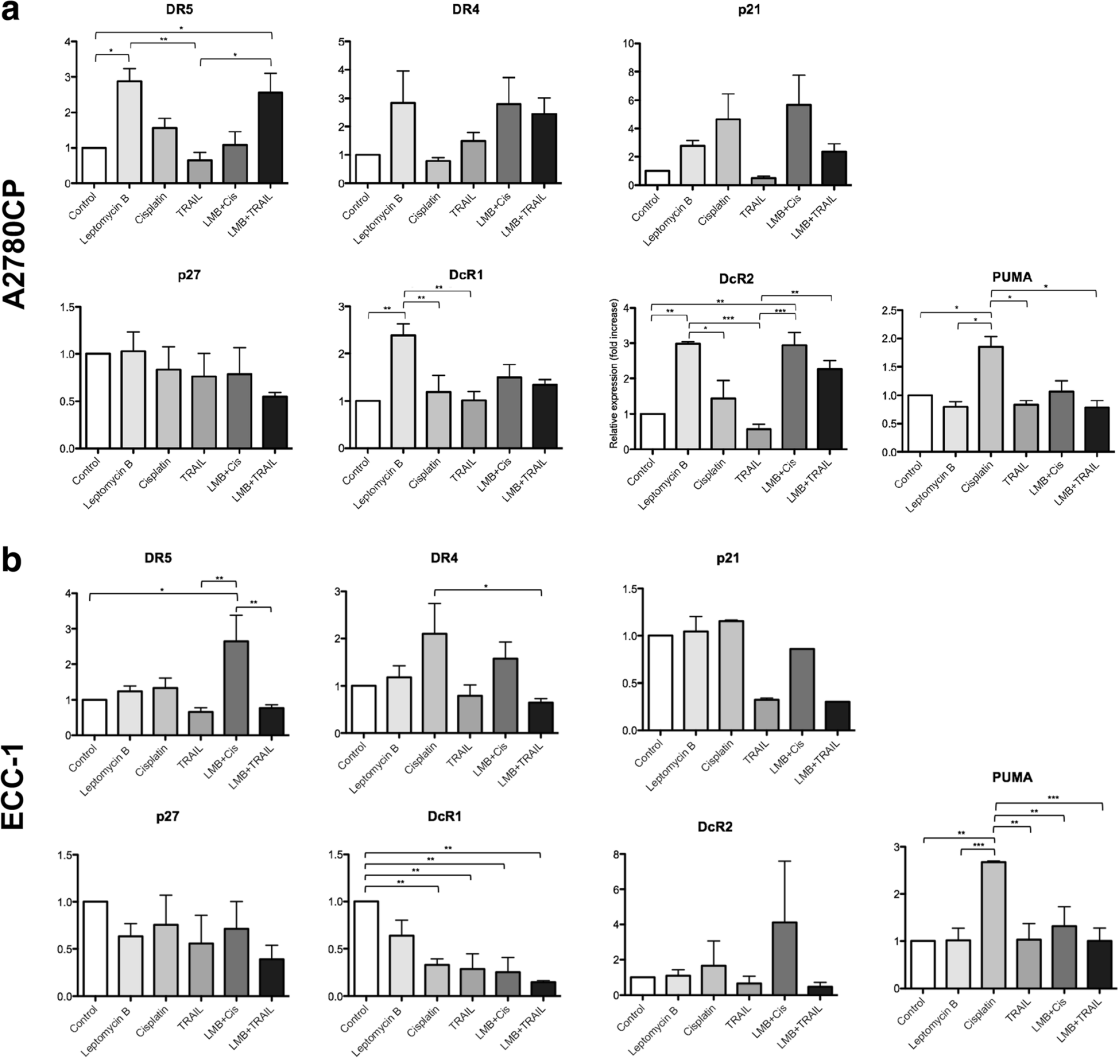
Leptomycin B, both singly and in combination with chemotherapeutic agents, modulates the expression of crucial apoptotic pathway genes in a cell-type specific manner **a** A2780CP and **b** ECC-1 cell lines were treated with leptomycin B (20 nM), cisplatin (10 μM), TRAIL (100 ng/mL) or a combination of leptomycin B with cisplatin or TRAIL for 24 h. They were then subjected to RT-qPCR analysis to quantify the mRNA expression of DR4, DR5, p21, p27, DcR1, DcR2 and PUMA. 18S mRNA expression was used as control for qPCR results. Results shown are representative of three independent experiments. Brackets are used to show statistical differences between treatment groups. All data are means ± SEM of three independent experiments. *, *p* < 0.05; **, *p* < 0.01; ***, *p* < 0.001

In order to determine the effect of the various treatments used in our experiments on the transcriptional landscape of the cells and further explain the observed sensitization effect, we performed qRT-PCR on A2780CP (Fig. 6a) and ECC-1 (Fig. 6b) cells. Cells were again treated with either LMB (20 nM), cisplatin (10 μM), TRAIL (100 ng/mL) or a combination of LMB with cisplatin or TRAIL. Cells were then processed for qRT-PCR analysis of TRAIL receptors DR4 and DR5, TRAIL decoy receptors DcR1 and DcR2, as well as p21, p27 and PUMA, pivotal proteins involved in cell fate. These proteins were selected based on the fact that they are crucial regulators of apoptosis and TRAIL response; alternatively, they allowed us to measure p53 activation, as p21/DR4/DR5 and PUMA are well demonstrated transcriptional targets of p53. The obtained results showed that the combination of LMB and cisplatin powerfully upregulated the expression of DcR2 in A2780CP and DR5 in ECC-1. Alternatively, the combination of LMB and TRAIL induced the upregulation of DR5 in A2780CP cells, but not in ECC-1; on the other hand, the same treatment produced a strong downregulation of DcR1 in ECC-1 cells, an effect that we did not observe in A2780CP cells. In any case, however, LMB treatments, either singly or in combination with cisplatin or TRAIL did not modulate the expression of PUMA, p21 or p27. Altogether, our results suggest that the combination of LMB with TRAIL sensitize the cell to apoptotic stimuli through the upregulation of death receptors expression and the downregulation of decoy receptors expression.

## Discussion

Tumor cell ability to resist apoptosis induction is a complex and multifaceted problem. Resistance to various cytotoxic agents is a fundamental hurdle to our capacity to treat these diseases and clinicians face such problems on a daily basis. While not one of the most studied, TRAIL resistance is one of the most widely described acquired resistance found in gynecological malignancies patients [17–19]. We believe it is imperative that we investigate novel methods that would counter this fatal mechanism and allow clinician to employ TRAIL-based therapies. Tumor cells can acquire resistance to apoptosis through multiple type of alterations, namely in the case of molecules involved in, or opposing, the apoptotic cascade. Considering the high amount of tumor suppressors acting as potential transcription factors, we hypothesize that the mechanisms regulating the localization of such factors could prove to be highly impactful molecular targets. The karyopherin B superfamily of nuclear shuttling proteins is an excellent example of such putative targets. Multiple published studies have shown that CRM1 inhibitor LMB is an excellent inducer of apoptosis presenting a surprisingly specific cytotoxic capability; various compounds mimicking its action, such as KPT-330, have been developed and are currently undergoing clinical trials (NCT02227251; NCT03095612). While we consider that generating new molecules targeting the nuclear-cytoplasmic apparatus is an immensely promising approach, we believe the general understanding of the involved signaling pathways is still underwhelming. The exact mechanisms by which these agents, both LMB and KPT-330, exert their tumoricide role is still largely unknown; it is our opinion that a deeper understanding of these regulation systems is required if we are to add what could be an exceptional tool to our molecular therapeutic arsenal. In this paper, we have sought to explore the clinical possibilities offered by interfering with CRM1-mediated nuclear shuttling. In that context, LMB is perfectly suited for fundamental research aiming to elucidate the role of CRM1 in chemoresistance establishment, to identify the potential chemotherapeutic agents for combined therapies, and to decipher the mechanistic role of CRM1 inhibitors in tumor suppression. It is well documented that caspase-3 levels are a powerful indicator of ovarian cancer prognosis as well as resistance to treatment and could act as independent marker for overall as well as progression-free survival [53, 55]. This information is compounded by the evidence that caspase-3 rapid turnover is a fundamental mechanism of acquired TRAIL resistance [53, 55]; the heightened levels of cleaved caspase-3 observed in our experimental context is thus highly relevant. As demonstrated by our results, TRAIL sensitization was brought in every cell line following LMB treatments, a profoundly useful effect considering the tumor-specific nature of TRAIL-induced cell death. Acquired resistance to cisplatin in A2780CP cell line was reversed, as was intrinsic resistance to cisplatin in SKOV-3 cell line albeit in a less spectacular fashion. The difference found between the ability of LMB to sensitize cells to cisplatin and TRAIL is intriguing. It is widely accepted that alkylating-like agents such as cisplatin act by cross-linking DNA strands, which cause intrinsic pathway-dependent induction of apoptosis. However, DNA damage is capable of activating the apoptotic program through multiple pathways [56], depending on the activation of multiple actors such as the p38-MAPK pathway and p53 [57, 58], both of which are regularly mutated in tumors. However, the extrinsic pathway is capable of operating some measure of cross-talk with the intrinsic pathway through Bid activation by caspase-8 [58]. It is possible that the studied cell lines present unshared mutations in those pathways, conceivably enabling LMB sensitization in a cell-specific manner; conversely, our results show that TRAIL sensitization does not seem to be dependent on cell line, underlining a chemosensitization mechanism that might be more fundamental than the one involved in cisplatin resistance, being that it is shared by all studied models. Thus, our results suggest that resistance to cisplatin and TRAIL are non-concomitant and emerge from separate molecular events in which CRM1 is involved. This is consistent with previous publications reporting that ovarian cancer cells that were resistant to TRAIL remained sensitive to other chemotherapeutic compounds [59]. In light of these results, we are allowed to think that LMB could potentially sensitize cells to a wide-range of death inducing agents, acting through both the intrinsic and extrinsic apoptotic pathways; these finding coalesce into a compelling treatment paradigm based on the disruption on nuclear-cytoplasmic transport. One major finding of our study is the synergistic nature of LMB, TRAIL and cisplatin induced apoptosis. As shown by the two-way ANOVA used, the concomitant use of LMB with either drugs significantly potentiate their action; therapeutically, this could not only aid in the prevention of chemoresistance, but could also allow therapeutic regimen to use lower concentration of chemotherapeutic agents. This, in turn, would greatly increase the quality of life of patients receiving such treatments. While as much as 50% of ovarian cancer cell lines are intrinsically TRAIL resistant [59–61], very little is known regarding the mechanisms enabling TRAIL resistance to be acquired in ovarian tumors. Earlier studies have linked caspase-3 degradation to this phenotype, but our understanding of this phenomenon is still limited. If we are to use TRAIL to treat ovarian cancer, it appears vital that we develop novel strategies capable of overcoming both intrinsic and acquired resistance to this agent. One of the mechanisms proposed in this paper is related to the modulation of the expression of both decoy receptors and functional receptors of TRAIL, DcR1/2 and DR4/5, respectively. Decoy receptors inhibit TRAIL-induced apoptosis through either competitive assembly with the dimer, dysregulating DISC assembly, or through inhibition of downstream caspase cleavage [62, 63]; however, it is also widely accepted that TRAIL decoy receptors do not only act as TRAIL-inhibiting receptors. While their main function appears to be the protection of normal cells against TRAIL assaults, their exact physiological roles remain obscure. The delicate balance between functional receptors and decoy receptors is also a fundamentally intricate equilibrium, a complexity that allows the exquisite specificity of TRAIL to arise in normal tissues; interestingly, the regulation of decoy receptor expression influence on TRAIL sensitivity is not only limited to the expressing cells but also key to the tumor microenvironment, and thus, general tumor susceptibility to this process of cell suicide [64]. The effect of our combined treatments seems to promote the expression of functional receptors and diminish the expression of antagonistic receptors. Moreover, data have suggested that DcR1 overexpression could enable TRAIL resistance to occur in endometrial carcinomas [65]. Considering that decoy receptors expression is mainly controlled by p53, and possibly NF-κB, an intricate, plurinodal network of regulation emerges [63, 66–68]; indeed, functional TRAIL receptors expression has been linked to the activation of multiple pathways, namely p53, NF-κB and ATF3 [69–71]. Taken together, these data suggest that the expression profile of both functional and decoy TRAIL receptors are dependent upon the same proteins, resulting in a system that possess rheostat-like capabilities in inducing cell death. Our results suggest, however, that the combination of a CRM1 inhibitor sensitizes the cells to TRAIL-induced apoptosis through the concomitant upregulation of functional TRAIL receptors and the downregulation of multiple inhibitors of the extrinsic apoptotic cascade, namely FLIP and the decoy TRAIL receptors. Our results show that p53 opposes this sensitization effect; both in the wild-type p53 cell line as well as mutated p53 cell line. These data suggest that TRAIL treatment somehow induces apoptosis in a p53 independent manner; it is possible that tumor cells hijack p53 transcriptional capabilities and, following p53 stabilization through nuclear accumulation, allow the abnormal expression of various cell-cycle progression inhibitors and apoptosis antagonists [72, 73]. The obtained results, while not significant, also show the ability of TRAIL to reduce p21 expression, even reversing its heightened expression observed in presence of LMB. While a canonical cell cycle inhibitor, it is also well demonstrated that p21 exert an anti-apoptotic effect through multiple pathway; mainly, p21 is capable of inducing the expression of a wide range of apoptotic inhibitors such as c-FLIP, XIAP and BCL-2 while also inhibiting caspases activation, either directly or through the inhibition of CDKs required for the full potency of the caspases cascade to be achieved [74]. Moreover, p53 could potentially upset the balance between TRAIL decoy and functional receptors; p53 inhibition could conceivably allow for the enrichment of functional TRAIL receptors and subsequent TRAIL sensitization. It is, of course, not excluded that the expression of functional TRAIL receptors, as well as their inhibitory homologs, might be modulated by post-transcriptional mechanism such as miRNA interference; considering the fundamental role of CRM1 in the export of miRNA, it is highly plausible that some measure of miRNA dynamic is altered following treatments [75]. Many groups have already reported positive, as well as negative effects of certain miRNA on TRAIL pro-apoptotic capabilities [76]; further experiments and future studies will certainly allow us to decipher the roles of such mechanisms in the sensitization effect of LMB to TRAIL. The results obtained in Figure 4c are also puzzling, considering the increased PARP cleavage with a drastically reduced cleaved caspase-3 levels. It is possible that, in that case, alternative caspases such as caspase-6 and caspase-7, take over the role of caspase-3 in directing the apoptotic program. Furthermore, the results obtained in the colony formation assay, while confirming the increased effectiveness of the combined treatment in the context of p53 knockdown, seems to show that this loss of caspase-3 cleavage does not reduce treatment effectiveness in ECC-1.

## Conclusions

Taken together, our results suggest that the combination of LMB and TRAIL synergistically induces apoptosis in a p53 independent manner and that p53 mutation/deletion could plausibly potentiate this effectiveness. We believe that the current overall low potency of synthetic TRAIL homologs represents the most critical hurdle to the success of TRAIL-based therapy; in that context, we anticipate that our results could, given time, form the basis of novel therapeutic strategies involving the targeting of nuclear-cytoplasmic shuttling mechanisms in order to sensitize tumor cells to the effect of TRAIL.

## Additional file

Additional file 1: **Figure S1.** Densitometric analyses of Figure 4a. Densitometric analyses of results obtained in ECC-1 B. Densitometric analyses of results obtained in A2780CP. Brackets are used to show statistical differences between treatment groups. All data are means ± SEM of three independent experiments. *, *p* < 0.05; **, *p* < 0.01; ***, *p* < 0.001. (TIF 1195 kb)

## Abbreviations

CRM1: Chromosomal maintenance 1
DcR: Decoy receptor
DR: Death receptor
FADD: Fas-associated protein with death domain
FLIP: FLICE-inhibitory protein
LMB: Leptomycin B
MTT: 3-(4,5-dimethylthiazol-2-yl)-2,5-diphenyltetrazolium bromide
NES: Nuclear export sequence
TRAIL: TNF-related apoptosis-inducing ligand
