# Supplementary figures and images for: Inhibition of CRM1 activity sensitizes endometrial and ovarian cell lines to TRAIL-induced cell death
Source: Cell Commun Signal. 2018 Jul 4;16:39. doi: 10.1186/s12964-018-0252-z (PMC6033231; doi:10.1186/s12964-018-0252-z)

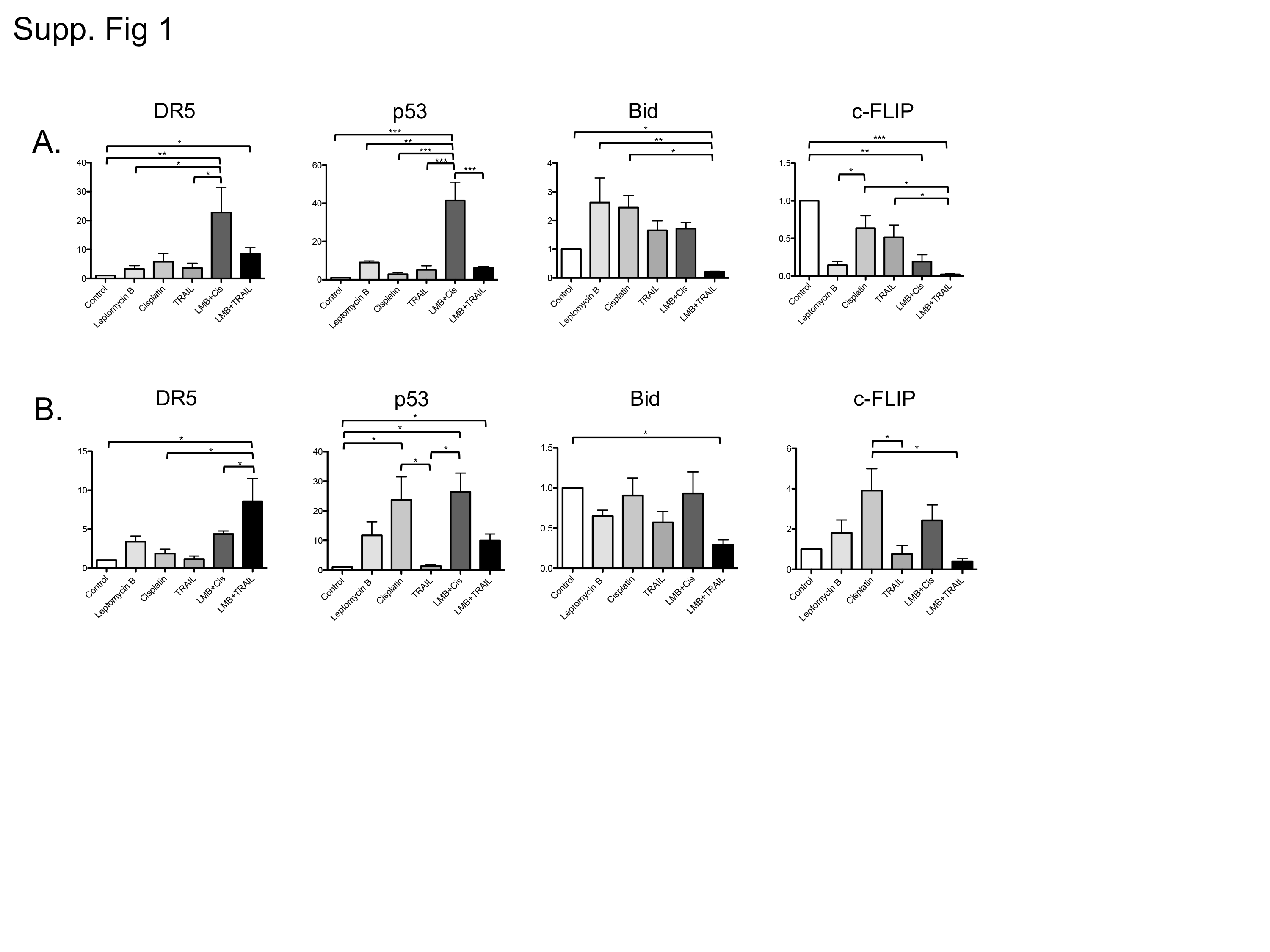

Supplement: Supplementary file 1 — Figure S1. Densitometric analyses of Figure 4a. Densitometric analyses of results obtained in ECC-1 B. Densitometric analyses of results obtained in A2780CP. Brackets are used to show statistical differences between treatment groups. All data are means ± SEM of three independent experiments. *, p < 0.05; **, p < 0.01; ***, p < 0.001. (TIF 1195 kb) [file 12964_2018_252_MOESM1_ESM.tif]
